# Naturalistic smartphone keyboard typing reflects processing speed and executive function

**DOI:** 10.1002/brb3.2363

**Published:** 2021-10-06

**Authors:** Mindy K. Ross, Alexander P. Demos, John Zulueta, Andrea Piscitello, Scott A. Langenecker, Melvin McInnis, Olusola Ajilore, Peter C. Nelson, Kelly A. Ryan, Alex Leow

**Affiliations:** ^1^ University of Illinois at Chicago Chicago Illinois USA; ^2^ University of Michigan Ann Arbor Michigan USA

**Keywords:** bipolar disorder, cognition, mHealth, trail making tests, typing dynamics

## Abstract

**Objective:**

The increase in smartphone usage has enabled the possibility of more accessible ways to conduct neuropsychological evaluations. The objective of this study was to determine the feasibility of using smartphone typing dynamics with mood scores to supplement cognitive assessment through trail making tests.

**Methods:**

Using a custom‐built keyboard, naturalistic keypress dynamics were unobtrusively recorded in individuals with bipolar disorder (*n* = 11) and nonbipolar controls (n = 8) on an Android smartphone. Keypresses were matched to digital trail making tests part B (dTMT‐B) administered daily in two periods and weekly mood assessments. Following comparison of dTMT‐Bs to the pencil‐and‐paper equivalent, longitudinal mixed‐effects models were used to analyze daily dTMT‐B performance as a function of typing and mood.

**Results:**

Comparison of the first dTMT‐B to paper TMT‐B showed adequate reliability (intraclass correlations = 0.74). In our model, we observed that participants who typed slower took longer to complete dTMT‐B (*b* = 0.189, *p* < .001). This trend was also seen in individual fluctuations in typing speed and dTMT‐B performance (*b* = 0.032, *p* = .004). Moreover, participants who were more depressed completed the dTMT‐B slower than less depressed participants (*b* = 0.189, *p* < .001). A practice effect was observed for the dTMT‐Bs.

**Conclusion:**

Typing speed in combination with depression scores has the potential to infer aspects of cognition (visual attention, processing speed, and task switching) in people's natural environment to complement formal in‐person neuropsychological assessments that commonly include the trail making test.

## Graphical abstract

This study explored the feasibility of using smartphone typing dynamics with mood scores to supplement cognitive assessment through trail making tests. Naturalistic keypress dynamics were unobtrusively collected from individuals with bipolar disorder and nonbipolar controls using a custom‐built keyboard and compared to serial administrations of the trail making test part B. Typing speed in combination with depression scores significantly predicted trail making test time and may have the potential to be used to assess cognition in real time to complement in‐person assessments.

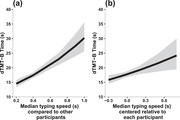

## 1 INTRODUCTION

Bipolar disorder is a mood disorder characterized by fluctuating manic/hypomanic and depressive episodes and often show both state (i.e., mood dependent) and trait (i.e., present during euthymia) cognitive deficits (Kurtz & Gerraty, 2009; Murphy & Sahakian, 2001). These cognitive deficits persist during periods of euthymia (Bourne et al., 2013; Mann‐Wrobel et al., 2011). Executive function, attention, verbal fluency, and memory are the most commonly reported impairments (Malhi et al., 2007; Osuji & Cullum, 2005). To assist in determining the nature and degree of cognitive impairment, a battery of neuropsychological tests is typically conducted (Yatham et al., 2010); however, these tests only capture a snapshot of the functional impairments at the time of assessment (Zulueta et al., 2020). Moreover, these assessments are conducted in a quiet controlled environment that bears little resemblance to modern work places or homes. Alternatively, self‐reports of cognitive difficulties may be considered in treatment decisions, but these are subject to memory biases and sometimes conflict with neuropsychological assessments (Burdick et al., 2005).

Connected technologies like smartphones and smartwatches contribute to disease monitoring and are more unobtrusive and granular than traditional methods (Hussain et al., 2019; Rashidisabet et al., 2020). This approach has the potential to evolve into a form of personalized treatment medicine that focuses more on predicting and preventing symptoms based on the individual (Flores et al., 2013). To this end, several recent studies have found that passively collected naturalistic smartphone typing dynamics may be associated with mood state and cognition (Cao et al., 2017; Lam et al., 2020; Mastoras et al., 2019; Vesel et al., 2020; Zulueta et al., 2018).

This current study determines the association between naturalistic smartphone typing dynamics and an adapted smartphone‐based version of the well‐validated Trail Making Test (TMT), an executive functioning measure of visual attention, processing speed, and set‐switching (Bourne et al., 2013; Yatham et al., 2010), in order to assess the feasibility of using typing dynamics to supplement traditional cognitive assessments. Traditionally, this test is administered using pencil and paper but has since been adapted to digital modalities albeit with some conflicting evidence of reliability between the original and adapted methods, likely due to variability in the mode of administration, device type, and comparison method (Drapeau et al., 2007; Fellows et al., 2017; Hannukkala et al., 2020; Latendorf et al., 2021). More broadly, previous studies using mobile phone‐based cognitive assessments have been validated in comparison to their respective traditional counterparts (Brouillette et al., 2013; Moore et al., 2017). TMTs have been found to be affected by repeated administrations through practice effect (Buck et al., 2008; McCaffrey et al., 1993). Bartels et al. (2011) found a significant practice effect when the TMT was administered frequently over a 3‐month period.

The objective of this study was to examine the relationship between keyboard dynamics and TMT part B (TMT‐B; administered both as a traditional pencil‐and‐paper test and serially self‐administered on a smartphone) in a group of participants consisting of both nonbipolar controls and adults with bipolar disorder. A secondary aim sought to determine if this relationship is modulated by mood symptoms and the practice effect on TMT‐B performance.

## 2 METHODS

### 2.1 Participants

The study participants consisted of individuals with bipolar disorder (*n* = 11) and nonbipolar controls (*n* = 8) with no personal or family history of psychiatric illness (see Table 1 and Zulueta et al., 2018, that used the same study sample) who were recruited by phone or email and already enrolled in the Heinz C. Prechter Longitudinal Study of Bipolar Disorder based at the University of Michigan (McInnis et al., 2018). To be included in the study, participants needed to use and have familiarity with an Android smartphone without any self‐reported impairments in fine motor skills or vision that would hinder TMT performance or keyboard usage, and for the individuals with bipolar disorder, frequent self‐reported mood fluctuations or previous longitudinal data suggesting rapid cycling of mood symptoms. Informed consent was obtained from all participants prior to inclusion in the study.

**TABLE 1:** Descriptive statistics of participants in the study

	Control	Bipolar Disorder	*p*‐Value
*n*	8	11	
Age (mean (SD))	46.12 (10.72)	47.09 (10.57)	.847
# dTMT‐Bs (mean (SD))	32.00 (16.05)	30.64 (17.11)	.862
HDRS‐17 (mean SD))	1.02 (1.49)	12.68 (7.80)	.001
YMRS^a^ (mean (SD))	–	6.00 (3.80)	–
Gender (% Male)	3 (37.5)	3 (27.3)	1.000

Abbreviations: dTMT‐B: digital trail making test part B; HDRS‐17: Hamilton Depression Rating Scale 17‐item; YMRS: Young Mania Rating Scale; SD: standard deviation.

^a^YMRS is not rated in nonbipolar individuals.

### 2.2 Data collection

Participants were issued a Samsung Galaxy Note 4 with a customized keyboard app installed, which they used as their primary phone over 8 weeks. This keyboard replaced the default keyboard and recorded every time a key had been pressed on the keyboard (termed a keypress event). All keypress events were tagged using the general category of keypresses (alphanumeric, backspaces, punctuation, etc.) and associated timestamps. Additionally, the timestamp of a system‐generated autocorrect event as well as when the user elected to select one of three suggested words were also recorded (tagged as autocorrection and suggestion). Actual text was not recorded. The keypress metadata was uploaded through the app to the study server hosted at the University of Illinois at Chicago using secure encrypted protocols.

**FIGURE 1:**
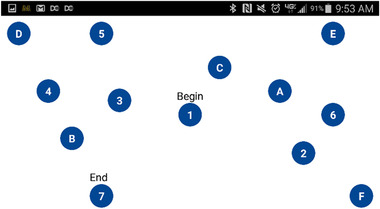
An example digital trail making test part B layout (one out of a total of 12 variations) deployed in this study

Participants took the pencil‐and‐paper version of the TMT‐B (pTMT‐B) at the beginning and end of the study. The digital TMT‐Bs (dTMT‐B) completed throughout the study were adapted to be completed on the participants’ smartphones through a separate research app that was downloaded onto the phone with the goals of collecting ecological momentary assessments of daily functioning and mood and included modified cognition tests (Ryan et al., 2020). The dTMT‐B consisted of alternating numbers and letters ranging from 1 to 7 (total of 13 circles) and respondents used their fingers to connect the circles in order, alternating between number and letter (see Figure 1). If participants connected the wrong dot, the blue dots would change color to red, and they would have to correct their error by going back to the last correct blue circle before moving on. In the morning and evening each day at preset times determined by each participants’ preference for days 1–17 and 45 through the end of the study, participants completed one of 12 variations of the dTMT‐B on the smartphone. The rationale for these two different time points was to potentially capture dynamic shifts in mood state in the bipolar illness verses one steady state of functioning. The time the test was taken, number of wrong moves, and total time of the test were recorded. A regression discontinuity design was used to account for the gap in recorded dTMT‐Bs in order to examine the effect of time at the beginning and end of the study separately. The first set of days was regarded as the first study period, and the second set of days was regarded as the second study period.

Research staff at the University of Michigan conducted phone interviews every week with the participants to administer the Hamilton Depression Rating Scale 17‐item (HDRS‐17) (Hamilton, 1967) and Young Mania Rating Scale (YMRS) (Young et al., 1978) following the Structured Interview Guide.

### 2.3 Data processing

**FIGURE 2:**
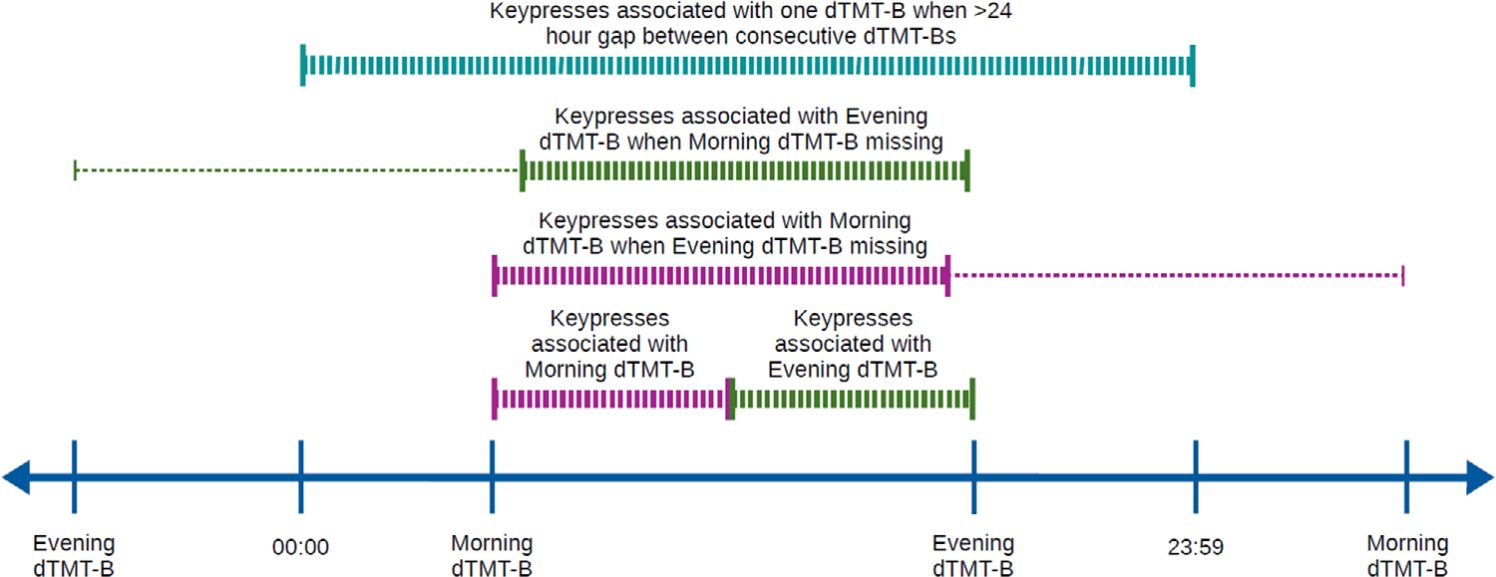
Schematic outlining how keypresses were assigned to each digital trail making test part B (dTMT‐B) to account for missing data

Participants who had completed the study and contributed at least 6 dTMT‐Bs and 20 keypresses per dTMT‐B were included in this analysis. Time windows to assign keypress events to dTMT‐Bs were created using the dTMT‐B timestamps such that each time window consisted of one dTMT‐B, one HDRS‐17 score, and multiple keypresses. This grouping allowed us to look at the relationship between each dTMT‐B and its proximal keypresses. The time between the morning and evening dTMT‐B was divided in half, and keypresses were assigned to a time window according to their timestamp. When there was only one dTMT‐B over a 24‐h period, keypresses during the respective half between existing dTMT‐Bs were assigned to the single dTMT‐B of that date. For gaps larger than 24 h between dTMT‐Bs, keypresses of the same date as the dTMT‐B of interest were assigned to that dTMT‐B (see Figure 2). Keypresses that fell outside the morning and evening dTMT‐Bs for days with two recorded dTMT‐Bs were omitted.

To calculate typing speed, the interkey delay (IKD), defined as the time lapse between two consecutive keypresses, was calculated across all keypresses within each time window. Median IKD was calculated for time windows with at least 20 character‐to‐character transitions of less than 8 s. The time cutoff of 8 s was previously defined by Vesel et al. as the end of a typing session (Vesel et al., 2020). Time windows that did not meet these criteria were omitted from the analysis. HDRS‐17 scores were backpropagated to the date of the previous recorded score and assigned to all dTMT‐Bs within the respective date range.

### 2.4 Statistical analysis

The intraclass correlations (ICCs) between the pTMT‐B and dTMT‐B were calculated to assess the consistency between the two modalities (ICC > 0.5 indicating adequate reliability) (Koo & Li, 2016). The first dTMT‐B for each participant was compared to their pTMT‐B taken at the beginning of the study, and the same was done for the last dTMT‐B and pTMT‐B at the end of the study. Additionally, ICCs and paired *t*‐tests were performed between the first and last TMT‐Bs for each modality.

Longitudinal mixed effects models (with maximum likelihood estimator (MLE) fitting) were used to predict dTMT‐B time (Singer & Willett, 2003). Forward‐fitted hierarchical models, compared with the likelihood‐ratio test, were built to examine the fixed effect of practice on dTMT‐B time, followed by the addition of mood ratings then typing metrics. Model 1 predicted dTMT‐B time from practice controlling for fixed effects of the time of day, age, and number of wrong moves on the dTMT‐B. Practice was measured in days since the start of each study period, respectively, and interacted with a discontinuity variable that accounted for the break (4 weeks) between sequential days of doing the trail making task. These three model parameters were needed to account for the practice effect. Model 2 added the weekly HDRS‐17 scores as level 2 (grand mean per subject) and 1 (weekly report; multiple reports centered within subject) variables along with dummy‐coded diagnosis (control vs. bipolar). Model 3 added typing speed as level 2 (mean of the median IKDs per typing session) and 1 (typing session at dTMT‐B time window; multiple windows centered within subject) variables. dTMT‐B time, number of wrong moves, and practice were log transformed in the models so that the residuals were more normal. All fixed terms were *z*‐scored to be able to compare the effects across different units. The random effects in the model included intercept per participant as well as the slopes of practice, the study period, and their interaction per participant.

A within group analysis of the individuals with bipolar disorder was performed to examine the effect of YMRS score on dTMT‐B time. Forward fitted hierarchical longitudinal models were constructed similar to the previous analysis in order to first predict dTMT‐B from HDRS‐17 score (Model 4) followed by the addition of YMRS score (Model 5).

All analyses were conducted in R (version 3.6.3) (R Core Team 2020). (See supplemental methods for details.)

## 3 RESULTS

There were no significant differences in age, gender, or mean number of dTMT‐B tasks completed between the two groups (Table 1). Individuals with bipolar disorder on average reported mild depression and mania symptoms (HDRS‐17 = 12.68, YMRS = 6.00), while the nonbipolar control group reported minimal depression symptoms (HDRS‐17 = 1.02).

To determine performance differences between the pTMT‐B versus dTMT‐B, ICCs were calculated to compare the consistency between the first and last pTMT‐B and dTMT‐B of the study (Table 2). ICCs were calculated between the first and last TMT‐Bs within modality, and all were adequate except the comparison between the last pTMT‐B and dTMT‐B. The first and last TMT‐Bs were compared within modality using paired *t*‐tests. There was a significant difference between the first and last dTMT‐Bs (*t* = 4.45, *p* < .001) but not pTMT‐Bs (*t* = 0.65, *p* = .52).

**TABLE 2:** Intraclass correlations (ICC) between the first and last pTMT‐Bs and dTMT‐Bs

TMT‐B	ICC	Confidence interval
Cross modality		
First pTMT‐B and First dTMT‐B	0.74	0.32‐0.9
Last pTMT‐B and Last dTMT‐B	0.14	‐1.38‐0.69
Within modality		
First and Last pTMT‐B	0.72	0.36‐0.89
First and Last dTMT‐B	0.68	0.29‐0.87

Abbreviations: TMT‐B: trail making test part B; dTMT‐B: digital trail making test part B; pTMT‐B: pencil‐and‐paper trail making test part B.

Forward‐fitted hierarchical longitudinal models were then used to predict dTMT‐B times from practice (Model 1), weekly HDRS‐17 scores (Model 2), and typing speed (Model 3) with each model building on the previous (Table 3). There was a significant improvement in each step, meaning that each successive model accounted for more variance than the previous. Model 3 was the best fit, so it will be further discussed in the subsequent sections.

**TABLE 3:** Model fits and significance of successive fits for the hierarchical models for all participants

	Deviance	Chi square (change in degree of freedom)	*p*‐Value
Model 1	92.63		
Model 2	76.61	16.02 (3)	.001
Model 3	47.05	29.57 (2)	< .001

Note:

Model 1: Age + log(# wrong moves) + Time of day administered + log(Day from start of each period) + Study period + log(Day from start of each period) : Study period.

Model 2: Model 1 + Diagnosis + HDRS‐17 (grand mean centered) + HDRS‐17 (subject centered).

Model 3: Model 2 + Median typing speed (grand mean centered) + Median typing speed (subject centered).

HDRS‐17: Hamilton Depression Rating Scale 17‐item; df: degrees of freedom.

Table 4 summarizes the effects of Models 1–3 of the individual predictors on dTMT‐B time in scaled estimates, where positive slopes indicate slower dTMT‐B times, and negative slopes indicate faster dTMT‐B times. Since estimates were scaled, they can be interpreted as effect sizes relative to each other.

**TABLE 4:** Summary of hierarchical longitudinal models for all participants showing the estimates and *p*‐values for the predictors of digital trail making test part B time

	Model 1		Model 2		Model 3	
Predictors	Estimates	*p*	Estimates	*p*	Estimates	*p*
Intercept	2.909	**<.001**	2.914	**<.001**	2.898	**<.001**
Age	0.113	**.036**	0.095	0.054	0.008	.784
log(# wrong moves)	0.222	**<.001**	0.221	**<.001**	0.220	**<.001**
Time of day administered	0.015	.146	0.016	0.111	0.013	.186
log(Day from start of each period)	−0.077	**.002**	−0.076	**<.001**	−0.069	**.004**
Study period	−0.055	**.013**	−0.049	**.032**	−0.039	**.044**
log(Day from start of each period) : Study period	0.032	.084	0.042	**.048**	0.041	**.045**
Diagnosis			−0.036	0.604	−0.180	**.001**
HDRS‐17 score (grand mean centered)			0.109	0.127	0.189	**<.001**
HDRS‐17 score (subject centered)			0.048	**<.001**	0.038	**.004**
Median typing speed (grand mean centered)					0.189	**<.001**
Median typing speed (subject centered)					0.032	**.004**
Random effects (variance)						
Residual	0.060	.058	0.058			
Intercept | Subject	0.049	.039	0.010			
Day since start of period | Subject	0.001	7.32e−10	0.0003			
Study period | Subject	0.001	.003	0.002			
Days since * Study period | Subject	0.001	.003	0.002			
Model fit						
Marginal *R* ^2^ / conditional *R* ^2^	0.389 / 0.663	.461 / .679	0.624 / 0.682			
log‐Likelihood	−46.317	−38.307	−23.523			

HDRS‐17: Hamilton Depression Rating Scale 17‐item.

The bold values highlighted the *p*‐values that were significant.

While significant in Model 1 (slower dTMT‐B with increasing age, *p* = 0.036), age was no longer a significant predictor of dTMT‐B in Model 3. The importance of age decreased as successive models were fitted, which suggested that age had shared variance with and was accounted for by HDRS‐17 score and typing speed.

There was an expected strong effect of the number of wrong moves on the dTMT‐B in all models. The more wrong moves a participant made on the dTMT‐B, the longer it took them to complete the task. This predictor had the largest relative effect size for predicting dTMT‐B compared to the other predictors, consistent with scoring of the dTMT‐B.

**FIGURE 3:**
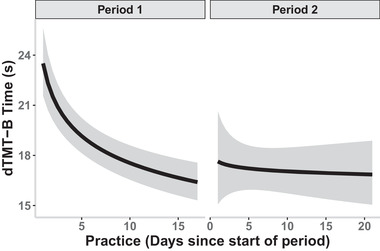
Digital trail making test part B (dTMT‐B) time as a function of practice in days since the start of the study period generated by Model 3 with ribbons showing the 95th confidence interval

Practice effect, modeled using days since the start of each period, was log transformed, since we expected participants to quickly improve on dTMT‐B before plateauing. As seen in Figure 3, Model 3 showed a significant effect of the day from the start of each period with participants speeding up on the dTMT‐B on each successive day they took it (*b* = −0.069, *p* = 0.004), suggesting a practice effect. This effect was seen in period 1, but not period 2. At the beginning of period 2, participants were faster on the dTMT‐B than at the beginning of period 1. This is shown in Model 3 through the significant interaction between the day from the start of the period and the study period (*b* = 0.041, *p* = 0.045). Finally, the time of day in which the participants completed the dTMT‐B had no effect on dTMT‐B. The relative effect sizes for these predictors were much smaller than that of the number of wrong moves as these predictors did not directly affect the total time of dTMT‐B completion like with making wrong moves.

Diagnosis was observed to have a significant effect on dTMT‐B (*b* = −0.180, *p* = 0.001). Individuals with bipolar disorder had faster dTMT‐B times than nonbipolar controls, but only when controlling for HRDS‐17 score and typing speed.

**FIGURE 4:**
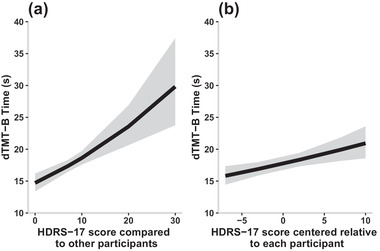
Digital trail making test part B (dTMT‐B) time as a function of grand mean centered (a) and subject centered (b) Hamilton Depression Rating Scale 17‐item (HDRS‐17) score generated by Model 3 with ribbons showing the 95th confidence interval

There was a significant effect of the grand mean centered HDRS‐17 score on dTMT‐B (*b* = 0.189, *p* < .001; Figure 4), which suggested that participants who were on average more depressed relative to each other took longer to complete dTMT‐B than participants who were less depressed. In addition, the subject‐centered HDRS‐17 score significantly predicted dTMT‐B (*b* = 0.038, *p* = 0.004), meaning that relative to their own weekly HDRS‐17 scores, when participants were feeling more depressed, they also took longer to complete dTMT‐B. The relative effect size of the grand mean centered HDRS‐17 score was larger than that of the subject centered HDRS‐17 score, suggesting that each participants’ overall mood compared to the other participants more strongly predicted their dTMT‐B than each individuals’ fluctuations in mood on their own dTMT‐B. On the contrary, YMRS score was not predictive of dTMT‐B (see Tables S1 and S2 in the Supporting Information).

**FIGURE 5:**
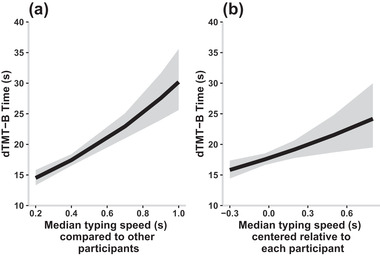
Digital trail making test part B (dTMT‐B) time as a function of grand mean centered (a) and subject centered (b) typing speed generated by Model 3 with ribbons showing the 95th confidence interval

As seen in Figure 5, there was a significant effect of the grand mean centered typing speed on dTMT‐B performance (*b* = 0.189, *p* < .001), suggesting that participants who on average typed more quickly completed the dTMT‐B more quickly than other participants. Additionally, the subject centered typing speed significantly predicted dTMT‐B (*b* = 0.032, *p* = 0.004), meaning that relative to themselves, participants who typed more slowly during one time window had a slower dTMT‐B time on that respective dTMT‐B compared to their average time. As with the HDRS‐17 scores, the grand mean centered typing speed had a larger relative effect size than that of the subject centered typing speed, which suggested that the participants’ overall typing speed compared to other participants was a stronger predictor of dTMT‐B performance than each individuals’ fluctuations in typing speed.

## 4 DISCUSSION

This study examined the feasibility of using passively collected smartphone typing speed and clinician ratings of mood to supplement formal neuropsychological assessments of select executive function domains. We showed that depression severity combined with naturalistic smartphone typing speed has the potential to supplement a person's dTMT‐B performance. Additionally, we observed a practice effect from frequent repetitions of dTMT‐B.

As there was adequate consistency between the first pTMT‐B and first dTMT‐B taken in the study, evidenced by the corresponding ICCs, our dTMT‐B was deemed a valid medium to assess executive functioning. We observed an improvement in the dTMT‐B at the beginning of each study period followed by a plateau in performance with a more drastic improvement and more gradual plateau in the first study period compared to the second. TMT‐Bs have been well‐documented to show a significant practice effect upon repeated administrations (Buck et al., 2008; McCaffrey et al., 1993) with one study suggesting a time of up to 1 year between assessments to remove the practice effect (Basso et al., 1999). Since an increasing number of assessments are being digitally adapted and administered more frequently, such as through ecological momentary assessments, it is important to understand and quantify the effect of repeated administrations. Future studies using repeated administrations of the dTMT‐B (and by extrapolation digital adaptations of other similar neurocognitive tasks) will need to carefully consider, and properly account for if indicated, practice effects in the analyses. Further, caution should be exercised when comparing performances of traditional in‐person neurocognitive tasks to those of their digital adaptations that are remotely deployed and administered.

Second, depression severity was associated with the dTMT‐B time at both the inter‐ and intrasubject level. Participants who were more depressed completed dTMT‐B more slowly than participants who were not depressed. This aligned with previous studies that found impairment in executive functioning in depressed patients with bipolar disorder (Kurtz & Gerraty, 2009; Martínez‐Arán et al., 2004; Ryan et al., 2012). This effect was stronger when each participants’ average depression score was compared to others than when within participant fluctuations in depression were used to predict their fluctuations in dTMT‐B. One likely explanation is the backpropagation of the weekly mood scores to the dTMT‐Bs during that week, which did not account for potential mood changes between the weekly assessments and decreased the granularity of the data for the intrasubject variability. Interestingly and somewhat surprisingly, YMRS, on the other hand, was not predictive of dTMT‐B, which might have been due to the moderate correlation between depression and mania scores in our study sample that frequently exhibited mixed features.

Third, typing speed was further associated with the dTMT‐B at the inter‐ and intrasubject level. Faster typers completed the dTMT‐B more quickly than slower typers. Moreover, although not as strong of an effect, participants’ individual fluctuations in typing speed reflected their fluctuations in dTMT‐B over the course of the study. These results suggest that cognitive domains measured by dTMT‐B, including visual attention, processing speed, and set shifting, are also engaged while typing on a smartphone and are potentially captured through a person's typing speed. While this study focused solely on typing speed as determined using the median IKD, future directions could investigate how other keypress measures, such as alternative ways of measuring typing speed, error rate, or typing variability, are related to specific aspects of TMT performance like errors made during task completion.

In this study, age had a significant effect in the first hierarchical model of dTMT‐B performance. However, once depression scores, diagnosis, and typing speed were introduced into the subsequent models, the effect was no longer significant. The change in significance may be due to the fact that typing speed explained the effect on dTMT‐B in place of age, especially considering the effect age had on typing speed reported by Vesel et al. (2020). Further, in our models, time of day was never a significant predictor of dTMT‐B performance. While it is unclear why this is the case, we note that Vesel et al. examined the relationship between time of day and typing speed, while this current study used typing speed as a fixed effect to predict dTMT‐B.

Fourth, a diagnosis of bipolar disorder was a significant predictor of dTMT‐B, though this effect was only seen after controlling for depression score and typing speed at both the inter‐ and intrasubject level. Due to the small sample size in this study, there were a small number of participants in each group, which limits the interpretability of the results. In partial compensation for the limited sample size, each participant contributed numerous observations over the course of the study, which increased the subject intravariability. These observations though varied in frequency due to the naturalistic approach of data collection.

There are limitations to the current study. Most importantly, contrary to traditional in‐person assessments, the environmental variables in which the dTMT‐B was collected could not be known. The remote administration of the dTMT‐Bs, although convenient for the participant, meant that the environment in which they completed the tasks most likely varied between tasks and participants. This confound might at least partially explain the higher variability in the dTMT‐Bs. Other possible confounds could include the lack of a formal neurological assessment of motor function in our study participants and potential subtle neurological soft signs that have been reported in those diagnosed with bipolar disorder, which might add variance to TMT and keyboard performance independent of depression severity (Sagheer et al., 2018).

Additionally, TMTs generally comprise of two parts: part A and B. Our study consisted solely of part B for the digital administrations, which meant that we were unable to separate processing speed from set‐shifting in our analyses. However, one may expect that the ability of set‐shifting is relevant in naturalistic typing (e.g., switching between QWERTY and special character layouts). Nevertheless, further work is needed to further replicate and determine the clinical applicability of these findings.

## 5 CONCLUSION

With the rise in smartphone usage, there has been an increase in mobile health apps looking to provide users with feedback based on constant monitoring. The present study examined the utility of the smartphone's keyboard as a medium to passively measure select domains of executive function when combined with periodic assessments of the participant's mood. The derived metrics collected in‐the‐wild did not place any extra time demand on the participant, thus providing a possible unobtrusive way to monitor changes in select domains of executive function at a higher granularity.

## CONFLICTS OF INTEREST

Olu Ajilore is a cofounder of KeyWise AI. He is on the advisory boards of Embodied Labs and Blueprint Health. Alex Leow is a cofounder of KeyWise AI, has served as a consultant for Otsuka US, and is currently on the medical board of Buoy Health.

### TRANSPARENT PEER REVIEW

The peer review history for this article is available at https://publons.com/publon/10.1002/brb3.2363

## Supporting information

SUPPORTING INFORMATION

## Data Availability

The data that support the findings of this study are available from the corresponding author upon request.
