# Supplementary material for: Naturalistic smartphone keyboard typing reflects processing speed and executive function
Source: Brain Behav. 2021 Oct 6;11(11):e2363. doi: 10.1002/brb3.2363 (PMC8613429; doi:10.1002/brb3.2363)
Supplement: Supplementary file 1 — SUPPORTING INFORMATION [file BRB3-11-e2363-s001.docx]

Supplemental Information

Naturalistic Smartphone Keyboard Typing Reflects Processing Speed and Executive Function

Mindy K. Ross^1^, Alexander P. Demos^1^, John Zulueta^1^, Andrea Piscitello^1^, Scott A. Langenecker^1^, Melvin McInnis^2^, Olusola Ajilore^1^, Peter C. Nelson^1^, Kelly A. Ryan^2^, Alex Leow^1^

^1^University of Illinois at Chicago

^2^University of Michigan

**Supplemental Methods**

Data processing was conducted in python (version 3.7.4)^1^ using the pandas package (version 1.2.0)^2,3^ and NumPy package (version 1.17.2)^4^. Statistical analysis was conducted in R using the tidyverse package (version 1.3.0)^5^ for data wrangling and visualization, and the lme4 (version 1.1-23)^6^ and sjPlot (version 2.8.4)^7^ packages for reporting mixed effects models. Assumptions were checked using the DHARMa package (version 0.3.2.0)^8^, and p-values were calculated using Satterthwaite approximations from the lmerTest package (version 3.1-2)^9^.

**Supplemental Results**

We used forward fitted hierarchical longitudinal models to examine the effect of YMRS score on dTMT-B in individuals with bipolar disorder only. There was no significant improvement in model fit between Models 4 and 5 following the addition of YMRS score, as shown in Table S1. This may be due to the correlation between the mean HDRS-17 scores and mean YMRS scores for the group with bipolar disorder (r = 0.54), indicating a possible presentation of mixed features in the participants.

Table S1. Model fits and significance of successive fits for the hierarchical models for participants with bipolar disorder only.

|  | Deviance | Chi Sq (change in df) | **p-value** |
| --- | --- | --- | --- |
| Model 4 | 50.43 |  |  |
| Model 5 | 47.79 | 2.64 (2) | 0.267 |
| Model 4: Age + log(# wrong moves) + Time of day administered + log(Day from start of each period) + Study period + log(Day from start of each period) : Study period + HDRS-17 (grand mean centered) + HDRS-17 (subject centered) + Median typing speed (grand mean centered) + Median typing speed (subject centered) | | | |
| Model 5: Model 4 + YMRS (grand mean centered) + YMRS (subject centered) | | | |

We present Models 4 and 5 as a comparison to Model 3, since they contain only individuals with bipolar disorder. This allows us to get an estimate of the changes in effect sizes relative to this group. As shown in Table S2, the patterns for the overlapping variables seen in Models 4 and 5 were similar to that of Model 3, which predicted dTMT-B for all participants. Relative to Model 3, there was an increase in the relative effect size for the grand mean centered typing speed; otherwise, the pattern remained the same with the exception that the subject centered median typing speed and study periods were not significant in these models.

Supplemental Table 2. Summary of hierarchical longitudinal models for participants with bipolar disorder only showing the estimates and p-values for the predictors of dTMT-B.

|  | Model 4 | | Model 5 | |
| --- | --- | --- | --- | --- |
| *Predictors* | *Estimates* | *p* | *Estimates* | *p* |
| Intercept | 2.952 | <0.001 | 2.954 | <0.001 |
| Age | 0.011 | 0.769 | 0.002 | 0.953 |
| log(# wrong moves) | 0.251 | <0.001 | 0.250 | <0.001 |
| Time of day administered | 0.001 | 0.949 | 0.001 | 0.930 |
| log(Day from start of each period) | -0.072 | <0.001 | -0.069 | <0.001 |
| Study period | -0.034 | 0.256 | -0.040 | 0.172 |
| log(Day from start of each period) : Study period | 0.057 | 0.044 | 0.055 | 0.053 |
| HDRS-17 score (grand mean centered) | 0.158 | <0.001 | 0.177 | <0.001 |
| HDRS-17 score (subject centered) | 0.057 | 0.003 | 0.054 | 0.005 |
| Median typing speed (grand mean centered) | 0.234 | <0.001 | 0.245 | <0.001 |
| Median typing speed (subject centered) | 0.024 | 0.125 | 0.023 | 0.136 |
| YMRS score (grand mean centered) |  |  | -0.042 | 0.109 |
| YMRS score (subject centered) |  |  | 0.004 | 0.824 |
| **Random Effects** | | | | |
| Residual | 0.062 | | 0.062 | |
| Intercept \| Subject | 0.004 | | 0.003 | |
| Day since start of period \| Subject | 5.34e-10 | | 6.69e-10 | |
| Study period \| Subject | 0.004 | | 0.004 | |
| Days since * Study period \| Subject | 0.003 | | 0.003 | |
| **Model Fits** | | | | |
| Marginal R2 / Conditional R2 | 0.714 / 0.732 | | 0.722 / 0.733 | |
| log-Likelihood | -25.216 | | -23.896 | |

**References**

1. Van Rossum G, Drake FL. Python 3 Reference Manual. CreateSpace; 2009.

2. McKinney W. Data Structures for Statistical Computing in Python. In: Walt S van der, Millman J, eds. Proceedings of the 9th Python in Science Conference. 2010:56-61.

3. The pandas development team. Pandas-Dev/Pandas: Pandas 1.2.0. Zenodo; 2020.

4. Harris CR, Millman KJ, Walt SJ van der, et al. Array programming with NumPy. Nature. 2020;585(7825):357-362.

5. Wickham H, Averick M, Bryan J, et al. Welcome to the Tidyverse. Journal of Open Source Software. 2019;4(43):1686.

6. Bates D, Mächler M, Bolker B, Walker S. Fitting Linear Mixed-Effects Models Using lme4. Journal of Statistical Software. 2015;67(1):1-48.

7. Lüdecke D, Bartel A, Schwemmer C, Powell C, Djalovski A, Titz J. SjPlot: Data Visualization for Statistics in Social Science.; 2020. https://CRAN.R-project.org/package=sjPlot Accessed December 14, 2020

8. Hartig F, Lohse L. DHARMa: Residual Diagnostics for Hierarchical (Multi-Level / Mixed) Regression Models.; 2020. https://CRAN.R-project.org/package=DHARMa Accessed December 14, 2020

9. Kuznetsova A, Brockhoff PB, Christensen RHB. lmerTest Package: Tests in Linear Mixed Effects Models. Journal of Statistical Software. 2017;82(1):1-26.
